# Inverting the ‘dementia triangle' as a salutogenic approach to avoiding the “treadmill of disasters” in changed behaviors: a qualitative study

**DOI:** 10.3389/frdem.2026.1891190

**Published:** 2026-09-08

**Authors:** Kasia Bail, Lara Wiseman, Georgina Chelberg, Nathan M. D'Cunha, Stephen Isbel, Henry Brodaty, Thomas Morris, Diane Gibson

**Affiliations:** 1 Centre for Aging Research and Translation, University of Canberra, Canberra, ACT, Australia; 2 ACT Health Directorate, Canberra, ACT, Australia; 3 University of New South Wales Centre for Healthy Brain Aging, Sydney, NSW, Australia; 4 HammondCare The Dementia Centre, Greenwich, NSW, Australia; 5 Dementia Support Australia, Greenwich, NSW, Australia

**Keywords:** behavioral and psychological symptoms of dementia, changed behaviors, dementia, health professionals, health services, healthcare systems, qualitative research

## Abstract

**Introduction:**

People with dementia are likely to experience changes in behavior, and health and aged care services are struggling to effectively respond to these behaviors alongside a growing aging population. This research aimed to explore the experiences and recommendations of clinicians involved in providing care to people with dementia, including people experiencing changed behaviors, across multiple settings including community, acute, transitional and residential care.

**Materials and methods:**

This qualitative study used semi-structured interviews with clinicians, analyzed using reflexive thematic analysis. A stratified purposive sample from all relevant care settings was used in a metropolitan region.

**Results:**

Dementia experts (*N* = 25) from various settings provided information regarding experiences and recommendations to enable access to effective care services for people with dementia and their care partners in acute, community and residential dementia care. The six themes identified were: (1). Workforce, gerontological expertise and team composition to respond to acuity of needs; (2). Creating enabling and dementia-friendly environments; (3). Appropriate prescribing in the context of physical and mental comorbidities, (4). Safety and occupational violence as indicators for risk management; (5). Early intervention and de-escalation maximizing non-pharmacological interventions; and 6. Leadership of innovative and communicative transitional care services. This last theme was key in determining suitable community responsiveness and decreasing stigmatization of people with dementia to enable accessibility to residential care and other services.

**Discussion:**

Dementia experts from across settings provided information regarding experiences and recommendations to enable access to effective care services for people with dementia and their care partners in acute, community and residential dementia care. Early intervention, de-escalation, and education can enable better community responsiveness, but need health service investment. A visual framework was developed to represent the recommendations, “the Salutogenic Triangle,” highlighting a broad base of community support services that focus on wellness, with additional targeted multidisciplinary transitional services, including clinicians with gerontological expertise to improve dementia care for all severity levels. This upstream/downstream public health policy approach is provided to guide policymakers, service providers and clinical leaders to positively and effectively impact changed behavior in people with dementia, the communities they live in, and the health services they deserve.

## 1 Introduction

Changes in behavior are experienced by up to 90% of people with dementia over the course of their disease and may include symptoms such as anxiety, depression, delusions and/or hallucinations and/or behavioral symptoms such as restlessness, disinhibition, and/or aggression (Brodaty et al., 2003; Dementia Support Australia, 2024; McShane, 2000; O'Connor et al., 2018). Individual disease progression can contribute to changes in behavior, which may vary by type, frequency, duration and severity depending on the type and etiology of the disease (Brodaty et al., 2003; Burns et al., 2012; Sampson et al., 2014) resulting in multiple care setting interactions over the course of the illness.

In Australia, of the estimated 446,500 people living with dementia most (66%) are living in the community. However, more than 50% of nursing home residents are living with dementia and up to 10% of hospital bed days are accommodated by people with dementia (Dementia Australia, 2026; Australian Institute of Health and Welfare, 2025). Primary, secondary, and tertiary settings each present clinicians with a range of challenges to implementing evidence-based person-centered interventions in responding to people experiencing changes in behavior.

Internationally, it remains common to refer to such behaviors as behavioral and psychological symptoms of dementia or the shorthand terminology of BPSD. In Australia, however, the Dementia Australia language guidelines state that the preferred terms are changed behaviors or expressions of unmet need (Dementia Australia, 2023), with use of behavioural and psychological symptoms of dementia restricted to clinical contexts only. Changed behaviors is the terminology used by Dementia Support Australia and state health departments. Alzheimer's New Zealand also uses changed behaviors in its materials. In this article, which presents exploratory qualitative research on Australian clinicians' experiences and recommendations on providing care to people with dementia across health, disability, community and aged care services, we have adopted the language of changed behaviors.

Within the academic literature and clinical practice, there is growing awareness that commonly used descriptors such as BPSD, “behaviors of concern,” “disruptive” or “challenging behaviors can frame both the behavior and by extension the person with dementia in a judgemental and stigmatizing way (Soofi, 2025). Simultaneously, such language risks failing to recognize the impact of external factors that may be contributing to behavioral expression (Cunningham et al., 2019; Dupuis et al., 2012; Swaffer, 2014; Westera et al., 2017). Alternative terminology such as “behaviors of unmet need” or “changed behaviors” (Dementia Australia, 2025) are informed by people with living experience of dementia. These terms recognize that behavioral responses are influenced by the care environment, and seek to interpret and understand behavioral changes from the perspective of the person with dementia who may be in pain, feeling vulnerable, unsafe, or angry and is seeking to communicate their unmet needs, or who may be experiencing impacts from past trauma (Atee et al., 2021b; Cations et al., 2024; Johnston et al., 2026; Warren, 2022; Westera et al., 2017; Woods, 2001). Recognizing the complex ways in which neurological, biological, social, care, and environmental factors may contribute to the expression of changed behaviors by people with dementia provides clinicians with opportunities to implement strategies to prevent or reduce the occurrence, frequency and/or severity of modifiable behaviors (Brodaty et al., 2003; Warren, 2022). Evidence on patient, carer and service outcomes highlights that first-line, gold-standard approaches of non-pharmacological interventions are key in managing behaviors associated with dementia as a modifiable and unmet need (Warren, 2023).

Australian research on the experiences of people with dementia, care partners and clinicians highlights the need for new approaches to dementia care (Borbasi et al., 2006; Burley et al., 2022; D'Cunha et al., 2025a; Kable et al., 2015; Low et al., 2021). The National Dementia Action Plan (2024-2034) identifies eight actions to improve the lives and care of people with dementia in Australia (Australian Government, 2024). The actions include promoting a human rights approach and tackling stigma associated with a diagnosis of dementia (Actions 1 and 2); improving dementia diagnosis, post-diagnostic services, treatment co-ordination and support for people with dementia and their care partners (Actions 4-6); and building workforce capability and promoting evidence-based innovation (Actions 7 and 8) (Australian Government, 2024). Low et al. (2021) identify a number of principles that should underpin health and aged care service models for people with dementia and argue that none of the existing models of service delivery meet them. Principles include: needs and evidence-based service delivery integrated across primary, acute and sub-acute health care, aged care and social services; the involvement of a multidisciplinary workforce; promotion of autonomy, social participation and rehabilitation; enabling individual care planning through supported decision-making; and promoting the health and wellbeing of people with dementia, their care partners and families. Other Australian research has focussed on mapping dementia services, finding a heavy reliance on general (rather than dementia-specific) services (Tabatabaei-Jafari et al., 2024). Research with clinicians from health and aged care servicesin the ACT identified services for people with dementia experiencing changed behaviors as a particular gap, with residential care providers reporting they lack the support, skills, and care environments to provide appropriate care, contributing to prolonged hospitalisations and adverse outcomes (D'Cunha et al., 2025b). Capturing the perspectives of clinicians working directly with this population is necessary to inform the development of coordinated, dementia-specific service models that can reduce pressure on the acute health system and improve quality of life for people with dementia and their care partners. Elsewhere, the higher rates of delayed discharges of people with dementia has come under scrutiny, with concerns that this affects hospital bed availability, avoidable decline and hospital-acquired adverse events (Aaltonen et al., 2021).

Brodaty et al. (2003) developed a seven-tiered triangular or pyramid model to assist in dementia care service delivery planning, specifically for the care needs of people with dementia experiencing changed behaviors. This Australian model is applied internationally, particularly when examining more severe changed behaviors. The model is valued for distinguishing between differing severity of symptoms, aiding understanding of prevalence and service needs, and often used for policy and planning purposes (Van Den Bossche et al., 2024). However, as Brodaty and colleagues warned in their original article, the model is problematic when (mis)used to define populations and design services. Warren (2023) highlights that service design and workforce education perspectives need to be changed from a focus on pathology to a focus on personhood, in relation to concerns about potential restrictions to humanhood by labeling people as static within tiers or definitions of behaviors (Warren, 2023). The model does not (and was not intended to) identify systemic and structural factors that have been shown to contribute to changed behaviors. That is, unmanaged pain (66%), environmental factors (58%), poorly educated staff (41%), and lack of personalized care practices (40%) (Westera et al., 2017). Consequently, there is a need for a nuanced understanding of expert clinician experiences on dementia care across all settings where care is delivered, to inform recommendations for constructive short- and long-term strategies to improve outcomes for people with dementia experiencing modifiable changed behaviors.

## 2 Methods

This study employed a qualitative methodology to investigate the experiences and recommendations of clinicians currently providing care to people with dementia, including people experiencing severe changed behaviors, in a metropolitan Australian city. This study was approved by the University of Canberra Human Research Ethics Committee (HREC 13851).

### 2.1 Recruitment

The research team sought to recruit a purposive sample of at least 20 participants. A stratified purposive sampling approach was used to collect health, community and aged care system and clinical care insights. The existing professional networks of the research team lead responding to the health services initiation of the research brief, supplemented by snowball sampling, was used to identify a range of clinicians. These included multidisciplinary team members (nurses, doctors and allied health clinicians) working within different health and aged care services and settings (such as acute, community, residential and transitional) and contexts (public, private) and involved directly in the care of people with dementia. This approach was used to include clinicians who were most likely to provide useful and important information as well as diverse disciplinary, health care service and setting perspectives (Robinson, 2014). A member of the research team (LW) sent email invitations to potential participants including project information and a consent form. Suggestions for additional participants were sought, and email contact was made through this process, with follow-up emails sent if no response was received to the initial invitation. Consent forms were returned by participants prior to the interviews, either via email (for online interviews) or in person on the day of the interview (for onsite interviews).

### 2.2 Data collection

The interviews were conducted by two members of the research team (LW and KB) between July and August 2024. Interviews were either 1:1 small-group (up to 3 people) and were conducted online (via Microsoft Teams) or in person (onsite at clinicians' workplaces), depending on participants' preferences. To promote reflexivity the interviewers made field notes and discussed the interviews with other members of the research team during regular meetings. Automated transcripts of interviews were generated and then reviewed and edited for accuracy through reference to the original recordings by two members of the research team (LW & GC) who also analyzed the transcripts.

### 2.3 Data analysis

The interview transcripts were analyzed using a reflexive thematic analysis approach (Braun and Clarke, 2006, 2019, 2021). Initial analysis of interviews occurred through a two-stage process involving two researchers (LW & GC). The transcripts were read and re-read with meaningful text segments initially descriptively coded using a deductive approach to identify case examples; current practice; and recommendations to enable improved service provision to address research aims. The results of this coding were tabulated in a Microsoft Word document to enable discussion between coders for consensus and then re-coded using an inductive approach to identify themes and sub-themes by two researchers (LW & GC). Two researchers (LW & GC) met to discuss coding and ensure the breadth of participants' experiences were reflected in the analytical approach. The themes were shared with other members of the research team (KB, DG, NDC & SI) to assess appropriateness and relevance and modified through further discussion and analysis (KB, DG & LW) until consensus was reached. Team member disciplinary backgrounds include nursing, social science, allied health, public health and health science, all with personal experience of dementia within their family. Reflexivity was conducted through ongoing touchpoints and reflective discussions among team members regarding potential unexamined bias, to maximize the voice and meaning from the participant contributions (Braun and Clarke, 2021).

## 3 Results

Of the 45 individuals invited to participate, 25 clinicians consented and completed an interview. The median interview length was 45 mins, with a total of 727 mins of interviews collected. Nine interviews were 1:1, five involved two participants and two involved three participants. Despite our best efforts, we were unable to complete interviews with general practitioners (GP) or community-based nurse practitioners (NP). Analysis occurred across the 25 participants, seven different disciplines, five settings and 3 funding types (see Table 1), and the experiences and recommendations of participants are presented. Specific roles are not presented to avoid reidentification of participants.

**Table 1:** Characteristics of participants.

Demographic variables	Category	n (25)	%
Gender	Female	23	92
	Male	2	8
Occupation	Allied health	4	16
	Medicine	2	8
	Nursing	18	72
	Paramedicine	1	4
Setting	Acute	12	48
	Community	2	8
	Community & Residential	1	4
	Residential	4	16
	Transitional^*^	6	24
Context	Public	17	68
	Public & Private	1	4
	Private	7	28

^*^Transitional settings were roles that worked across acute, residential and/or community settings, most often an outreach service from a public service into residential care/community homes to prevent avoidable hospital admissions but also included ambulance (emergency response).

Six key themes were identified: (1). Workforce, gerontological expertise and team composition to respond to acuity of needs; (2). Creating enabling and dementia-friendly environments; (3). Appropriate prescribing in the context of physical and mental comorbidities; (4). Safety and occupational violence as indicators for risk management; (5). Early intervention, and de-escalation maximizing non-pharmacological interventions; (6). Leadership of innovative and communicative transitional care services. These themes are outlined below with supportive quotations illustrating the concepts. Any editing for readability is provided using […ellipses…], and further supportive quotation examples are cross-referenced using (P#) in text and provided in Table 2.

**Table 2:** Themes with additional supportive quotations.

Theme 1: Workforce, gerontological expertise and team composition to respond to acuity of needs	
A man with dementia was admitted to [the] geriatric unit, sexually inappropriate behaviors, grabbing female staff. [We] didn't even have a special for this patient. He was discharged and readmitted at Older Persons Mental Health, a 1RN to himself, 1:1 ratio managing him. [Whereas it's a] 1:4 in geriatric unit.	P1
In mental health, when you have patients who are having similar levels of behaviors, they are younger and are often able to provide their own personal care [but in acute geriatric wards staff] have to do personal care on top of that. Yet [mental health] also have a 1:2 ratio! … And if you have someone in ICU [intensive care unit] or HDU [high dependency unit] setting who's requiring restraint, they have a 1:2 ratio as well. Whereas [acute geriatric wards…] meet all of those demands with the 1:4 ratio.[…] a lot of that is due to the fact of people not valuing the specialist knowledge [that] is required to look after people with such advanced pathophysiology.	P5
I think in ED… there has to be adequate staffing and safe staffing levels. […] I think they have to have appropriate staffing and most hospitals probably don't have appropriate staffing. I know that we don't have when I've requested a special for a patient, I'm often told, we can't get one, we can't get one.	P25
I think the ratio of staff and residents as well, because we can be right away there. We can provide them one on one engagement.	P9
It's managing triggers before they escalate and having a skilled workforce that is able to recognize those triggers and really know the residents extremely well and can almost anticipate when these behaviors are gonna happen. To have that diversional therapy type interaction and put [it] in place. So also staffing numbers in, I think we need you know, we need a greater representation of care workers in that space.	P4
There is no way that we can have geriatricians everywhere… knowledge transformation and building up the knowledge is the best way forward […]. If we can build up that knowledge in ED, that this is what we need from you when patients come, and then if they are aware of it and they understand, then our task will become much easier.	P13
Definitely need a […] specialized occupational therapist who can, you know bring in activities which are tailor made for each and individual patient	P3
There's a stroke nurse, a cancer nurse, a trauma nurse - there should be geriatric nurse … for clients who have dementia.	P21
The OT failed that person on their shower assessment because ‘that's not how you routinely shower'. They [allied health] just don't have that understanding… people with cognitive impairment revert to what they knew.…She grew up in a drought, a rural [property] and so you never ever had the shower running while you were soaping yourself, [but the OT] kept failing her, she was like, ‘no, she can't go home'.	P5
And I would also say, you know that one of the things that leads to that [aggressive behavior] in all environments […] is the occupational deprivation. You know, we are often very quick to put in behavior management plans that include how we communicate with somebody, but often I think that we forget that that they, this cohort of patients often don't get to engage in meaningful behavior or the meaningful behavior that they engage in is not what people think is appropriate for them and we don't have the, you know, they don't have alternate options and so that then leads to again the frustration and the outbursts and aggression.	P16
And so it's a, you know, it's training and supporting our nurses to understand communication partner-training and all the facets of that that you know are communication is a loop. And it's not just about the person […] with the impairment, it's about their partner as well and how that's facilitated. […] I think in terms of delivery of information, you know, chunking and check-in all of those kinds of language-based strategies would be, you know, helpful. […] All of those kinds of basic things that we don't probably get time enough to do or put, you know, enough emphasis on they're very simple, but very effective.	P23
When we get involved with a patient, we can see there's a communication impairment and we'll try and support them with that too. [Speech pathology] rarely get referrals on hospital wards for communication for a patient with dementia	P23
From my experience, there is usually 6 month lag between […] refer a patient to neuropsychology and we get any, any kind of significant news […] which is sometimes not helpful. I would [like to] see that to be much lower, I mean couple of months, 6 weeks, 8 weeks. […] I understand, I'm not criticizing a service that is simply because they don't have the staffing. Actually it will be best if we can have an MDT with neuropsych every week, flag patients to them, discuss with them.	P16
What I would love to see is a dedicated dementia support Allied Health Unit that has some allied health FTE that can create some programs that can be implemented by a family and care support team. That would keep people maximized in their functional capacity and minimize the challenging behaviors they experience so that they can stay in their community where they want to live for an extended period.	P16
The introduction of the [allied health practitioners] advanced practice model would marry very well with having some people who are like, you know, dementia experts essentially in their field.	P14
The first thing we asked when someone has an unmet need or displays a new […behavior], we ask […] UTI [urinary tract infection]? Pain? All of the clinical stuff we look at - was it too hot, too cold, his pants too tight […] It was the light, too bright!	P2
I think really when we‘re doing really good assessment, we're getting to build rapport with patients. We‘ve got to build trust with patients. We've got to make them feel safe. I don't know that we can do that without really taking time to get to know them, and I don't know that the environment of a hospital gives us that luxury, I suppose.	P23
We like to use less is more if that makes sense? […] To try and gain the person's trust using either staff members that they might be more familiar with or comfortable with, or family members that they might be more comfortable with to engage with them on our behalf. […] just going slowly, doing one thing at a time, not overwhelming them, overloading them and just speaking calmly, reassuring them that you're there to help them.	P15
I think we do it really well, is that we make connection with our residents. We get to know them we get to know their story. We create a connection based on trust and respect. […] We create a calm, settled environment […] we sit here and its calm as, and the reason its calm as is because we‘ve got the place settled. And that's because we create a connection to those people, we've created an environment where they feel happy and at home.	P9
There's not enough pain relief. […] Like most behaviors, unmet needs, are from pain, unmanaged pain.	P2
We like to consider what the cause of their behavioral disturbance might be, whether they've got an underlying infection, whether they're in pain or, you know, simply whether they're hungry or need to go to the toilet.	P15
I also collaborate with Dementia Training Australia to ensure my [RAC] staff are well trained in the dementia and they are aware of, you know, why resident display certain types of behavior of concern in order to identify [and] manage the trigger and also able to deescalate it on time.	P18
I can tell you a wonderful strategy from a previous home […] we do a 24-h monitoring of a resident's sleeping, waking, rising. What their day normally looks like, and we did this on all of the residents and we actually changed the cleaners and the meal services. So no, a cleaner would not be able to just go into somebody's room because they may not get up till 11 or they may be awake ‘til later at night. So we changed cleaning rosters, we trained our cleaners about, you know. But they weren't to go and […] disturb anybody. […] meal times was really important because some, some people like to awaken, get up later and stay up later at night. So to you know, we would change the meal times to 100% suit, their needs.	P4
It is good though, that there's recognition that the population is aging. At least that's been identified as priority. […] I think from an inpatient perspective, I think the things that will work well like is people's understanding of things like falls and delirium. […] Champions for delirium and things like that.	P21
I'm gonna say there needs to be a geriatric nurse in the ED […] all the time. Delivering specialist assessment of those clients and optimizing their care while they‘re stuck there for however long they're gonna be stuck there.	P21
Promoting the screening, whether it's fall screening, delirium screening, (early intervention) and if the screening is positive, then you put some strategies in place.	P17
Somebody who's there [in ED], ready, who has extra like studies in gerontology or they have a masters in dementia. Just imagine the difference that would make [.…] I think we're having a tsunami now and it would be really good to be more proactive.	P2
Well we see carers stress identified during the admission, but not actually addressed, because the patient themselves become medically stable and there's that pressure to discharge. And the carers are like ‘Oh, they‘ll be right'. So often when we go, it's like that chance to delve into that a bit more and when they're back home and you can you see them in their home environment and you see that it's not working.	P19
There's often questions around what might be driving a behavior and trying to think about what elements might be related to environments, what might be related to medication, what might be related to [a] particular neurological profile attached to dementia, and when differentiating those helps with respect to knowing how to work with that [person] and support someone.	P8
15.6-7.4,-13.6498ptYou know, clinically, they're very acute and they need very specialized care from nurses with intensive knowledge around how to provide that care and the underlying reasons and pathophysiology of why those presentations are happening.	P7
Theme 2: Creating enabling and dementia-friendly environments	
When I look at our ward environments and aged care environments […] getting away from these tiny, locked environments which actually just exacerbate all of their symptoms and going toward big open places where you can have you know where you can replicate little villages […] like the dementia villages type situation and even being able to do that in a hospital environment. You look at this new building […] they‘ve got it for the kids in the pediatrics section having big open spaces with lots of entertainment and things so people can engage with outside, so they don't feel like they're locked in. They‘re still locked in, but they don't ^*^feel^*^ like they're locked in because that's part of the problem.	P7
What we do have is a garden on each side. Each area has a garden, and we use the garden as a de-escalation space.	P9
And you know, having better spaces on the wards, the [acute aged care] ward has now got a nice little outdoor space that they're accessing […] having their morning tea and the patients are sitting out with families […] it potentially is something that helps to prevent [responsive behaviors…] from developing.	P23
So we do find [names residential aged care home… manages] behaviors quite well […] they can have the physical space to move, they can take themselves out of a space that feels trapped. […] So they allow residents […] to walk around the facility and not limiting the space. […] So there would be [a] facility that can cater to the need of the escalation of behaviors by just letting them walk around.	P24
I think quite often it comes down to the physical environment as well. The way the building is, designed so you can see in facilities where they're very closed in. It's a small unit; the lighting's not great. The door's very locked. We do find that behaviors aren't managed as well, […]. Whether it's just that that facility is, is it the chicken or the egg? I guess in that situation is that because they [residents with dementia] feel very trapped	P24
It feels like a home for them, because they can get inside the kitchen, they can help. You know, they can still - there's some residents who like help us with washing, we just let them do it because they feel like they're involved they, they sweep, but with the staff with them, like feel like home for them	P11
But we really do know that that person only eats, you know, apples in the morning, and they‘ve always had porridge before bed and that that little, tiny stuff, you know, and the cat, their cat […] wants to come along in the house or get the car. We'll try and mimic the life they had before as much as possible on entering our [care environment…]. I think a lot of the people with dementia that move into our houses, you can see this instant like ‘oh my goodness' because it's not any different to home.	P2
[Referring to RAC environments] They are not very well designed for dementia living and dementia purposes. You know, dead ends, […] no nice sensory gardens outside. There's not, you know, a nice environment to lead the residents seamlessly to locations or areas that they would enjoy and relax […]. We're not invested in doing this well.	P4
We are very careful about how we allocate people to say, the geriatric special care unit because you can't have two personalities or two people who are going to trigger each other and make it worse. […] I think it just comes back to [staff] ratios, and the model [of care] and the infrastructure and the environment in which these people are being provided care. […] if it was big enough that you could have somebody not have to come across the guy that triggers in and big open spaces and the ability to go outside and have one staff member to support that person, then you could achieve much better things I think.	P7
We then come back to having to put someone in a single room because they're aggressive or having to put single someone in a single room because they have COVID, and we have no other single rooms available for them.	P5
If there is one person escalating, it is very hard to segregate them from others. So the ideal unit should be that there should be some level of segregation. There be some level of flexibility about patients wandering because people will wander that is there, you know they, […] used to walk in their life, they were policemen, they used to do a lot of exercise. They would like to walk around, but here we don't have that space, so we restrict them and then we say that […] they are having problems. Actually, we created that problem because obviously that is their normal behavior and they want to do it, but I'm host at a hospital. We can't allow that to happen.	P13
I think that I think if you've got people like that [with dementia in ED] they actually need to be in a more secure area. They need to have a good area where it's calm, it's quiet. Maybe some really nice music playing […]. I don't think we do it really well, in terms of making them feel a little bit more settled. I think they need a specific dedicated area that they can't escape out of that is secure for them, where everything's a little bit more shut down where it's not these buzzers going, voiceovers are going, emergency buzzers are going, staff are running everywhere. That just agitates them some more.	P25
If they've got any sort of cognitive impairment, no matter what it is, they should be in a safer, less stressful environment is my opinion.	P26
I'd love to see a four-bed dementia specific ward in ED.	P2
So there is the ED environment does not meet geri's [geriatric patient's] needs for sure.	P17
15.6-7.4,-13.6498ptI'm just thinking of a person who we've only just discharged a couple of weeks ago, he was in this in the bed for 200 plus days, and without the use of any psychotropic medication that BPSD settled down and, the reason for the delay in discharge was waiting for nursing home placement.	P12
Theme 3: Appropriate prescribing in the context of physical and mental comorbidities	
If we can't get it right with our psychosocial model, because it's so loving and so gentle, it's such a safe place to fall apart. Then you obviously don't just have dementia. […] Like, you've obviously got a long trauma history […] when I dig a bit deeper, there's childhood trauma, domestic violence, undiagnosed mental health stuff.	P2
Doctors are so uneducated about dementia. I'm sorry, the prescribers, they're either over prescribing or under prescribing. There's no middle ground. There's not enough pain relief.	P2
Straight away the first instinct from the junior medical officer was like ‘let's just prescribe some benzos and some antipsychotics. […] it was actually [a nurse who said], ‘I think we need to ask the Cognitive Impairment Clinical Care Coordinator to have a look at this because I'm not real happy with going straight to antipsychotic medications' […]. They were able to identify […] significant pain and an underlying infection and that was treated and I think that really avoided having to use other pharmacological interventions.	P7
Falls […] they've had quite significant fractures and the only way to manage them and their pain and to transport them safely was to use give them some sedation to help with that.	P15
[describing challenges of implementing medication recommendations received from DSA, including need for pain] sometimes our GP says no, doesn't need pain relief, doesn't need this […] sometimes they don't allow us to, you know, work on it. You know, it may not work, but let's try for 3 weeks, 4 weeks. If it doesn't work, yes, happy to take it off.	P18
When I have [someone with] the psychotic symptoms, I need a toolkit.	P2
I think that antipsychotic drugs in this cohort of people [with severe responsive behaviors] is […] important because you need to be able to titrate that, you need to be able to see how it affects people	P9
Some kind of prescribing protocol between a few SILS [supported independent living services] in Canberra that are willing to do above and beyond.	P2
I think [medication prescribing] consistency in meeting these more severe responsive behaviors could be beneficial. It needs to be tailored to the individual patient.	P5
This is a difficult one, isn't it? Because you want to have these consistent guidelines and sort of standard approaches so that everybody has the same access to care, but you also don't want to restrict [it] so much that you then can't have people being responsive to what the person needs. I think at the moment, it's really just like if you get […] this geriatrician, you're probably going to start on [medication X], whereas if you get this geriatrician probably [medication Y], you know, they just choose their preferred drug of choice. And that makes it very difficult for the nursing staff as well, because there doesn't seem to be any standard consistent approach to how they actually manage it.	P7
So there's a bit of a conflict in there with that one as well [intersection between mental health/older person psychiatry and geriatric services] it's really good if we can also talk to each other [geriatric services, GP and OPMH].	P22
[responding to changed behaviors] is shared work and we really need to be organized in a way that either we‘re not doubling up or […not] leaning on one bulk of a team vs. another […] building ties between our services [mental health and geriatrics] and we're going to have a joint case conference, for example on a patient.	P12
[aged care team] have some issues with older persons mental health […] some frustration sometimes that they will not step in sooner than they do, so they‘ll keep saying no, ‘It must be a delirium' […] despite the fact that they've got a long history of mental health issues.	P24
15.6-7.4,-13.6498ptWe just cannot deal with […] all of the psychiatry and BPSD. We're happy to, […] provide a consultation service, a consultation to that higher rung [severe behaviors] but not actually taking the referrals for all of those [less severe behaviors].	P12
Theme 4: Safety and occupational violence as indicators for risk management	
So we see very aggressive physical behaviors at times, so they'll grab you, they'll punch you, they'll scratch you, they'll spit on you. They'll yell at you. They'll throw things, they'll accuse you of stealing or holding them against their will.	P25
[describing a hospital ward that accommodates people with dementia experiencing changed behaviors] one of the highest occupational violence rates in [health service].	P3
[I got punched in the face] it was a patient with the oxygen tubing who, he had wrapped [it] around his throat. And because I was trying to take the tubing away from his throat, he thought I was choking him, but he was choking him. And, you know, he just [demonstrated punching motion] straight out.	P26
I think that if someone‘s, you know, very tearful or very upset […] as a clinician, […] you're in a safer space to comfort somebody. Whereas I think if you have somebody who's very aggressive or angry or physical, I think that's probably the most challenging for most of our clinicians […] often our [staff] are sort of young, female, 22 year old and they might be faced with a man who's in his 60s, very well built, very strong, very angry.	P23
My team is predominantly from Nepal and India, […] the ones that hit the most for them are the racial comments. […] that is what they find the hardest. […] you can rationalize the aggression, you can rationalize the other things, but when, when it's about your culture, to my team, that's what they find the hardest.	P5
I can say that there have been instances where somebody was punched or somebody was attacked without any kind of aggravation from a patient who have [been] diagnosed with dementia on the ward and not only the patient, the staff has also been, you know, have received injuries and they have gone off the work etcetera in last few years.	P13
Because what people with dementia are doing with their unmet needs or behaviors, it's just trying to tell you something that they can't verbalize.	P2
Patient gets aggressive when he becomes incontinent of feces. Staff needed wards person support to change his pad to ensure staff safety. If staff change the patient's pad as soon as he becomes incontinent, we can minimize the responsive behavior. […] If we lodge for wards person through DHR, the job it's more than half an hour, even if [we] code it as an urgent job on DHR, it will not be quick we can't leave the soiled pad for 30mins, it makes it worse, they will be hurting themselves and hurting other people	P1
I think my experience has been that the behaviors that we see are related to someone needed to communicate something and the environment is not conducive to that, or the interactions that they're receiving are not conducive to it.	P23
You know, like the staff are afraid to go […] to the resident because [the resident] tries to hit with the belt.	P18
We've had three women with terrible behaviors, and one of the big things that they did was throwing things, like throwing things at people and hitting out at the staff and actually hitting staff. And that was very difficult. Very difficult and staff were scared of them.	P6
Sometimes we have to wait to be able to get the help, that's where it's difficult. When you've got someone that's suddenly behaving totally uncontrollably, that help is not there and then only, the only thing we can do is call the ambulance. And that's not their job […] we don't want to do that. But when we can't control them, that is the last resort that we have.	P6
Occupational violence training, I don't think [RAC staff] get any … so quite often they may respond to a situation and make it worse because they didn't know how to de-escalate.	P24
It's getting that behavior before it ends up that they're at the level of, you know, so that diversional therapy type situation is much more needed in our homes and also our, I'd say, our staff could be a lot better skilled.	P4
At the end of the day, it's got to be about patient safety. […] and it's got to be about the safety of the nurses. […] you want to make sure your nurses are safe.	P25
We have instances where we wanted two wardsmen urgently to ensure staff and patient safety, due to responsive behaviors, but a Code Black brings seven to ten wards people, plus security staff, ten people coming into the room. That can be a traumatic experience for the patient	P1
15.6-7.4,-26.3498ptWe have done this this this, this this [implementation of non-pharmacological approaches] you know we have followed up with DSA, Geriatrician, we have done the medication changes. We have taken our like residential medication management review. We have involved everything as we could do. [But behaviors continue]. Now we are in a state where I don't know what to do next in that situation	P18
Theme 5: Early intervention and de-escalation maximizing non-pharmacological interventions	
I really think the early intervention and engagement is key […] so we have a reduction in the pointy end of what we see on the acute care of the elderly wards. That would be my number 1.	P14
So many years ago, the Memory Assessment Service was set up in our geriatric outpatient area, and there was some dedicated allied health at the time, which over time has been swallowed elsewhere. And I remember when that was set up, I thought this is a great opportunity for MDT early intervention with the support of geriatricians, very similar to the model that they use in the […] UK.	P16
There is a lot […] to be done in community actually. […] I see a [lady] who is 70 plus herself and now the husband is developing dementia. […]. He never had an ACAT. You know, he's still driving […] there's no EPOA appointed and […] now he's having behavioral problems, and he will never want to leave the home […]. Now, suddenly they come to me in the clinic. And now what can I do? You know, suddenly to solve all these problems?	P25
I remember seeing this woman who was multilingual […] and her particular dementia was affecting her language and so her ability to spell, her ability to talk to her grandchildren, all these things were changing and so she really wanted to write letters to her grandchildren before she lost the ability to do that. So to know that this is a primary progressive aphasia like that hits your language first also means she knew on a bucket list what to triage. (P8)	P8
You know, there are 70 different causes of dementia, potentially, and […] in their end stages they might present very similarly. But in the early, in moderate stages, they might be quite different […] so I think early diagnosis and early differential diagnosis is really important.	P8
Unfortunately, I don't think we have a very robust MDT clinic. […] We would like neuropsych input. We do have a social services person, a social worker who can quickly assess these patients. So we can refer them from the [memory assessment] clinic. Sometimes actually they are in the in the clinic [and] available, if the staffing is good, and we can refer them straight away if there's any significant concern. […] we do refer lots of patients to social worker[s] from that clinic because often there are social issues highlighted. […] I can say that neuropsych people do prioritize our referrals. […] neuropsychology assessment basically is a detailed assessment of somebody's dementia subtype or cognitive impairment subtype, as some patients come in with a primary progressive aphasia as well.	P13
The main […] catalyst for the creation of SPICE was the fact that there is very little MDT, multidisciplinary care approaches for people with dementia in the ACT. […] wanted to try and address a gap for people with dementia at all stages […] to have an integrated approach to dementia care in the ACT. […] what we did was have […] a look at known evidence-based practices that support […], maximize the health, wellbeing and quality of life of people with dementia. […] we started probably with an early intervention phase because we wanted to test the program and we wanted to test that with a cohort of patients that were probably in a mild to medium moderate […] dementia. […] It was just the recognition that there was not any significant […] allied health-led [programs] so people can certainly get access to medicine. […] but as we know, the benefit of non-pharmacological treatments for people with dementia is significant […].	P16
[…] biggest challenges […] in that area are people who don't have support, who don't have those networks, so it can be very difficult and can be difficult to engage with and difficult to know the history of how they've gotten to that point in their life and if they are then assessed to not have capacity to be able to make their own decisions, that then opens a whole other range of complexities in needing to support people with applications for guardianship or management in the community. […] so that can be quite a drawn-out process with the end goal really being to make sure that people have the appropriate supports to either stay in their home or move to residential aged care and in the process preventing an admission to hospital.	P14
But then a barrier to us keeping people at home can often be that because we are the first people suggesting services, there is a wait time. So sometimes reasons for people coming into hospital if it's for behavioral management or psychological management, it's because the services can't be there in time to prevent that.	P21
[role of DLN discharge liaison nurse in identifying potential patient needs on discharge] there are many, many times when I‘ve walked in, when I've read the notes and it's gone “patient is safe,” that that the doctors have written, you know, “well supported,” and I walk in there and that's just a load of bollocks. […] I come out and go well, […] they've undiagnosed dementia. They‘re this. They're that. They‘re driving […] and the doctors go, “Oh, my God!,” and they just suddenly go into this panic, “I didn't know,” and I say to them, you have to spend the time, I said I understand, but don't tick, don't say that they're safe for discharge unless they send a DLN where we can spend that bit of time and look through stuff and go, well, actually, no, this is really bad. And I‘ve had so many patients where […] their wife's been caring for them at home. But you know what? The wife's having chemo. So what's happening? Or, she's got COVID […] you look at the whole situation as a bigger picture.	P25
[describing role of transitional care team] we offer a timely and short-term holistic approach, […] part of the assessment is looking at obviously the physical assessment as well as medication review, […] link carers as well as patients to any appropriate external resources, […] so we're kind of the link if you want. […] facilitating that transition from hospital to home. […] so looking at what […] has been identified as concerns, if you want, on the ward whether it's related to social needs, whether it's related to medical needs, or getting referrals from the community and […] refer to get them safe as long as possible where they are, in linking them to appropriate resources and avoid any ED presentation or hospital admission	P17
We refer to ADACAs, sometimes when people need help with supported decision making. Care Finders when people don't have any resources or support people to help them and with linking them to services,[…] so GPs, home care providers, ACAT, Dementia Australia […] community social workers […] referring on to other allied health or medical specialists as needed to make sure that patients have a plan, a safe plan of treatment moving forward..	P19
I also don't know if that's because we‘re not picking up or providing good enough psychoeducational supports to people in those mild stages so that we're not preparing them, and therefore the wheels fall off. Rather than there being a, you know, a kind of future planning that will enable that to happen in a more successful, less reactive emergency acute driven way.	P8
I think we need to have like you know we have really great people who have like a heart failure pathway. And once you get diagnosed with a heart failure, you have, you know, these are your next steps. These are what you need to do. There is an XY and Z. […] think we need a dementia diagnosis pathway or a cognitive impairment diagnosis pathway that like GPs can access, you know as soon as this kind of like oh I'm thinking there might be the start […] of a cognitive impairment here, like they should be starting them on these pathways starting those conversations, starting looking at you know is there an enduring power of attorney in place here? Do you have loved ones? Who can make decisions for you? […] starting those conversations really early so that the wheels aren't falling off and we're not ending up with these people massively in trouble in hospital.	P5
Because the nurses in the facility are usually new, so sometimes they don't know the resident quite well, or sometimes they're just agency nurses […] they don't really know what to do.	P22
[…] we do find that the people with behaviors, even the not severe behaviors, there is a very wide gap in the management of those people. […] we find there's not a lot of experience in the facilities and in terms of the staff knowing how to manage. We do get a lot of phone calls in crisis situations, asking us to come out and assess someone who [is] suddenly aggressive or more aggressive than normal because they're just at a loss of what to do.	P24
I can think of multiple examples off the top of my head of people who were in homes [residential aged care] and have been sent to hospital because of their behaviors and their care managers have called the families and said we can't take your mum back, your mum or your dad has to go to SDCP [Specialist Dementia Care Program]. There's nowhere else who will be able to manage your mum, but actually, once we‘ve just put in some, […] behavior management plan. You've got them on a range of medications. It's working for them, and you know, everything's kind of settled down. They haven't gone back to the home that they've come in from, but we‘ve gotten them to another facility that wasn't an SDCP, [we] have been able to manage their care needs.	P5
Sometimes it is because they're just so frustrated with not being able to have their needs met or get, can't communicate well […]. One of the things that leads to [aggression] in all environments […] is the occupational deprivation […] we don't have the […] alternate options and so that then leads to […] the frustration and the outbursts and aggressions.	P16
I think that a lot of the communication behaviors that we see are from either boredom or frustration.	P23
One of the things […I] would like to see is some increased allied health specialty within the inpatient setting, particularly from an OT perspective and potentially even some psychological or speech pathology. Maybe I think our social work colleagues provide right support to people with dementia already, but I think there's some areas for improvement again in that therapeutic space.	P16
Did we trial the non-pharmacological measures as we described on behavior support plan like music or provide them whatever they like? For example, it could be something like an ice cream, something very small like an ice cream, but that could calm down and things like that.	P1
We absolutely need diversional therapists on the wards to support people, volunteers to help people have their meals.	P23
It is a sub-acute ward. It's got a little bit of lower, lower stimulus in one sense, in terms of it's not an acute ward with bells and buzzers going off every minute, but it actually is a higher stimulus ward with the occupational engagement program and access to some exercise physiology for maintenance of capacity.	P16
15.6-7.4,-13.6498ptWe are trying our best to identify the triggers and we communicate by completing this behavior chart [in the DHR]. It's easily visible for doctors and other allied health and everybody.	P1
Theme 6: Leadership of innovative and communicative transitional care services	
The other area that […] could be optimized as well is within the community care teams which are based in the community health centers. […] we already have our […] social work, physio, but they‘re not, they're more separate so they don't collaborate a lot as an MDT. And certainly, if you were to look at provision of specialized dementia care in that area, there would be room for improvement.	P14
I think that you know the support for in the community would probably benefit from being bolstered up […] including, you know, in our aged care […] for people who have those higher needs and having staff that are well supported to be able to deliver that.	P23
We try to sort out things our RADAR team does a fantastic job actually […]. They deal with lot of cognitive impairment in community. […] GP has referred me say for example memory assessment services but based on the referral [… I] thought this patient is struggling. […] let's do it at our home visit, don't do a clinic. Clinic will take 3 months' time, […] we differentiate that in the triage, that this patient needs a RADAR community geriatrician straight away. […] So you know concerns regarding their welfare, concern about their, you know, management of foods etcetera. […] The RADAR team tries to work with whatever connections they have outside in community.	P13
I think it's just all, you know, supporting people just to have a break and remembering the carers in the whole process. So if a patient is at home and is challenging, then it's equally challenging for that carer. So having enough support in the community for them as well so that they are, you know, able to take them home [from hospital] if they if that's an option for them from a medical point of view	P23
[…] my recommendation for government […] fund one of these [community-based day respite centers] in every, in every council area. What a difference it would make […]. It takes the pressure off […] health [system] more than I can ever articulate because half the people we meet are avoiding hospitals because of the advice we're giving them for free. […] people want more than a brochure. They want communities back, and when you've got more eyes on the prize and [the] carer is meeting other carers and you know DA [Dementia Australia] does some really good stuff and they've got support groups.	P2
I think there is a lack of awareness that all focus is about patients and patients' complex health care needs, and behavioral management and I think we tend to 1. not identify and 2 not address, […] that the carers' needs are as important, and as essential as patients' needs. […] trying to find some ways, having those difficult discussions with them as well. Because there is a lot of guilt, with ah, not being able to manage the behaviors, lack of awareness how to, and many of them would, will put themselves at risk to avoid admission to hospital or […] they don't ask, they're not really willing to ask for any help and think that [they] can manage themselves. […] have that discussion with them, saying that everyone has limitations and there is a lot of external resources out there. There is a lot of expertise that they can get and we're here to educate and make them aware of the most appropriate responses. But it's kind of planting seeds and it's a big process. It does take time.	P17
Well, we see carers' stress identified during the admission but not actually addressed because the patient themselves becomes medically stable and there's that pressure to discharge, and the carers are like ‘Oh, they'll be right'. So often when we go, it's like that chance to delve into that a bit more and when they're back home and you can you see them in their home environment and you see that it's not working	P19
We see people that are needing that either want to go into care or they're needing a level 4, but they haven't had anything initiated, ever, and that's where it's at that stage where we need something within a week or within a few weeks, and we know the reality is months waiting.	P19
[NP role in RADAR…] could provide some education across settings. […] the need for Geri's nurse in ED, the NP could assist with that. The NP could assess patients [who] are more at risk of being readmitted to hospital […] looking at the […] medical conditions […] working with hospital in the home, home based anything that could assist in preventing some presentation. […] Looking at identifying cognitive impairment or dementia a little bit earlier, whether part of a memory clinic having the NP doing some consult in that field.	P17
We need to look at the entire health structure, including the GP, including the community and the hospital, and we need to look at what, what can be done to improve at various different levels. Just one level will not work. […] I think we tend to focus a lot on the hospital here and everything is to be provided by the hospital. I think unless we change that, focus back to community, we will not be able to improve the entire situation. Because the hospital cannot on itself do everything. It has to be outside as well, which is lacking.	P13
[responding to changed behaviors] is shared work and we really need to be organized in a way that either we're not doubling up or […not] leaning on one bulk of a team vs. another […] building ties between our services […] have a joint case conference, for example on a patient.	P12
The challenge is […] resourcing and that ‘business as usual' is already so challenging. Being able to innovate, […] I think takes a certain amount of drive that I'm not sure if it's there at the moment, but it would be great to be able to see.	P14
And having that collaboration altogether […involvement of multidisciplinary team in how to best provide care for a particular person] it was a lot of time and effort for everyone, but […] the reason for that is this patient has frontal temporal dementia. You don't want him to come to ED […] He's very mobile. He won't do well in a secure environment. So we really need to work all together […] across disciplines to meet their needs […] I'm not asking for going above and beyond. I'm just asking for some flexibility to better manage their special needs.	P17
Mental health services are very keen to collaborate with geriatric colleagues […] we're not co-located […] both of our services are quite you know, distributed amongst [different hospitals in different geographic locations…] it would be wonderful whether we can actually co-locate […] you know the geriatric unit, but also the old age psychiatry units […] I think that would go a long way [toward better] collaboration and understanding where each other's challenges may be, but also in those where we need both our input, I mean […] we have a lot of patients where we share care.	P12
No nursing homes [will accept him] …Even though patient has been completely settled for months, you know, and there is no further suicidal ideation. When patient was having this delirium at that time [the] patient displayed some suicidal ideation….so now it is extremely hard to find a suitable [home] for this patient.	P1
They‘re not going to take those people who are tricky or those people who are under the public guardian because they don't have loved ones to care for them, or who've had previous responsive behaviors, because those are the people that are gonna be harder to meet the care needs.	P5
Delirium is probably quite a struggle […]. Someone who might be a bit combative or not let you assess them properly […]. Often if we can't do a proper assessment, then [paramedics] need to transport patients to hospital because we can't guarantee that they're gonna be safe in the nursing home or at home.	P15
[…] there's a lot of rejections. It's not uncommon to get […] 20 to 30 rejections.[…] we do a lot of our MDT, our multidisciplinary team here do an excellent job our Ots, our psychologists, our nurses, our doctors, we all work together. We put together a behavioral management plan. We stabilize people and sometimes it's still, you know, it doesn't suit. The answer back is it doesn't suit.	P12
There's a big variation in the middle there, and we're having a lot of people who aren't SDCP suitable but aren't MSU suitable and they just sit in the system and an acute hospital ward is not where these people should be living their lives.	P5
So people who have significant responsive behaviors, who are very difficult to place, who may require specialist dementia care program, the process for accessing that is incredibly convoluted […] like 12 steps.	P7
I think [SDCP] has made a difference […]I do think we are successfully getting these people out [of hospital], but I think there's significant improvement to make, to make it a more equitable and accessible program.	P5
If you ask our GP's in Community, they will say RADAR is one of the best teams in this hospital.	P13
[…] they're really, really good, like they're always happy to come out and assess a patient if they think it's appropriate for them and their skill set. […] I've always had really positive experience with GRACE.	P15
[One case] started with several phone calls to […] ambulance about a gentleman who had become aggressive […] the [ambulance] said no, they weren't gonna go out, they didn't think it was an appropriate transfer. [So in-reach service nurse] went out there and got hit quite quickly by this resident, so ended up calling the police […] they wouldn't come in unless he was actively hurting people and by that stage he calmed down […] it just shows the lack of resources for facilities to be able to escalate.	P24
The RADAR model could certainly be optimized through additional allied health funding […] inclusion of broader allied health teams to be able to provide more of that prevention, and a very targeted and comprehensive prevention.	P14
[would be good to have additional allied health staff within GRACE team…] OT, Physio	P20
[…] recruit a cognitive impairment clinical care coordinator […] get someone that's really passionate, the idea is that they help [GRACE team] with people with BPSD and […] assist with providing education to [residential aged care] facilities […for] those residents that they're having difficulty managing.	P24
Older person mental health […] as well and I think that team needs to be bolstered and we need to work better together as well [refers to relying on interpersonal connections between staff] I think we need to go beyond personal connections and try to have that kind of regular part of the structure.	P13
I just think it's about resourcing it [RADAR] correctly.	P3
[need to] work really closely with the aged care facilities because we have to build relationships and partnerships with them to assist in the transfer.	P3
[…] we now say you cannot have a respite with us unless you've seen a doctor within 33 months. You've seen a geriatrician. […] we got caught out. […] I just need the full picture and I think a lot of families in crisis for one aren't telling the full story because […] they've been burned, care burned for so long.	P2
We can't take residents that wander, being that we‘re so close to the road. It'd be awful if one got out and got run over or something […] we just don't have the facility to be able to reach out to look after people that are bad wanderers, [or] that call out a lot. Even now our rooms are all single rooms with ensuites, it's still difficult […]. You know if you've got someone that is singing out all the time. […] if the dementia increases and becomes worse, we do have to ask the family if they can take them somewhere else because we just can't manage them anymore.	P6
[…] staffing in this area is a constant challenge and coming up with solutions to like that sort of upskilling existing staff.	P4
Sometimes we have to wait to be able to get the help, that's where it's difficult. When you‘ve got someone that's, that's suddenly behaving totally uncontrollably, that help is not there and then only, […] thing we can do is call the ambulance and that's not their job. Do you know, and we don't want to do that. But when we can't control them, that is the last resort that we have	P6
Another challenge that we face is getting accurate and reliable information [from RAC staff when an ambulance has been called].	P15
I think that building relationships […] rather than them seeing us [the hospital] and Dementia Support Australia […] as you know, a threat we want to work with […] the facilities to facilitate a […] a sustainable transfer so[…]. What we find that if we have those behavior management plans in place, we do have success.	P3
I'm transitioning someone in a couple of weeks to a home. You know, they've previously had a bad experience in a home. How can I work with you now to make sure that this goes successfully for this person going forward […] if I actually just put, you know, a week in to making sure that this transition or you know, I have extra support to make sure that this transition goes well, then this will likely be a longer success rate rather than them just bouncing back to me in 2 weeks.	P5
[people experiencing changed behaviors transitioning from hospital to residential care] they‘re always going to be worse for that first couple of weeks because they're transitioning between environments and they‘re not familiar with the staff and they're not familiar with anything that's happening, and it's always going to be a high stress time, whereas if they just give them a little bit longer, they'd probably settle in.	P7
I think if we could have […] a similar team to the PACER team, which is the mental team that has the police, the ambulance and mental health nurses on it. If you had something like that for people with dementia, I think that would be amazing. So you have police, ambulance trained in dementia care or severe behavioral response	P24
[…] to get that shared understanding within other teams about like when it's not like those other courses of action that should be taken.	P12

### 3.1 Workforce, gerontological expertise and team composition to respond to acuity of needs

There were many participant comments regarding the quality and quantity of the workforce to respond to the needs of people with dementia, with the underlying principle that **staffing needed to be consistent with care needs**. For example, nurses working in acute settings highlighted the need to ensure nurse staffing ratios met the often complex care needs of people with dementia experiencing changed behaviors, particularly to develop and implement individualized behavior support plans and non-pharmacological interventions that require time and expertise:

*[The] acute hospital environment, is not where you wanna be when you‘re having, you know, responsive behaviors […] it just it always makes things worse, especially in a very small environment like our wards, where they are confronted with other people who are having similar issues and not having the right ratios to be able to look after these people. They really need 1:2 so that you can be there encouraging them to drink, making sure they're going to the toilet, encouraging them to shower and having somebody who's very skilled and specialized at doing that to prevent further complications (P7)*.

Participants highlighted the disparity between mental health wards and other high dependency areas which were more likely to have a 1:2 registered nurse staffing ratio for patients with complex behavioral care needs, compared to acute geriatric wards where nursing ratios were lower at 1:4 and described how this affected the quality of care staff could provide, and/or intensified the pressure on staff to maintain quality (P1, P5). Lower staff-to-resident ratios were also identified as an issue in nursing homes, and in emergency departments (P25), where the understanding of what is ‘adequate' for staffing knowledge was dependent on staff having skills and expertise in dementia, delirium and other complex geriatric conditions. Similarly, participants from residential settings identified how higher staffing ratios support the delivery of personalized and timely responses to expressions of unmet (P9, P4). Leading clinician champions across services were recommended to help upskill staff in multiple areas (P13, P3, P21). A dedicated champion/leadership role was seen as pivotal for cognitive care by enabling comprehensive assessment and patient histories to inform the development of behavior support plans to address patients' unmet needs and reduce escalations, “*[…] we could not survive without that role” (P3) (P23)*. The specialized skills of gerontological nurses in particular were identified as being crucial for effectively supporting people presenting with changed behaviors (P21).

*You know, clinically, they're very acute and they need very specialized care from nurses with intensive knowledge around how to provide that care and the underlying reasons and pathophysiology of why those presentations are happening. (P7)*

The need for appropriate skills and knowledge in caring for people with dementia also applied to allied health clinicians, who didn't always have sufficient education to be able to provide evidence-based care for this population (P5). Additionally, allied health clinicians with dementia-specific knowledge were identified as an often under-utilized element who could provide specialist interventions to improve rehabilitation during hospital or nursing home care. For example, occupational therapists to implement behavioral support interventions to address occupational deprivation (P16) or speech to facilitate better communication, particularly with people experiencing aphasia (P23). The importance of dementia-friendly multidisciplinary allied health teams was emphasized, including recommendations for access to multidisciplinary care teams at the time of diagnosis (P13), establishing post-diagnostic rehabilitation and reablement programs for people living in the community (P16) and encouraging dementia-specific advanced practice (P14). The need for additional training to reframe the scope of reablement, rehabilitation, and functional assessments within the context of dementia was identified as crucial to optimizing the effectiveness of the allied health workforce. Participants expressed there were often deficits in staff knowledge and skills around fundamental care for people with dementia such as building trust, through person-centered assessment, pain management, and minimizing invasive monitoring and procedures (P2, P23, P15, P9, P2, P15, P18), leading to preventable complications and admissions. Participants across settings identified the need to improve the **gerontological expertise and awareness** for all staff, not just clinicians (P4). A key recommendation was the concept of ‘Code Grays' with a single trained wardsperson to attend, rather than Code Blacks which signal a large security team response to aggressive behaviors:

*[The patient] was getting triggered by the wardsperson. …. the wardsman would hold him [when code blacks were called] and that made it worse. […] We started to call one wardsperson, they would come and observe in the background. We would tell them “don't grab the patient. Please talk to him, this is what he likes to talk about”. The regular wardsman learned how to deal with him and so [then] we could do personal care with him. (P1)*

At a broader level, clinicians working in acute settings observed that as older people make up the majority of in-patients, there is an increasing need and demand for gerontological, dementia and delirium expertise across the hospital system (P21). Examples included the importance of gerontological nursing expertise in the emergency department (ED) (P21, P17, P2), as well as in the role of discharge liaison nurses in understanding and taking into consideration the broader social context of a person's presentation. This related to referrals to other support services as well as identification of an accompanying care partner's fatigue and/or state of crisis (P19). Participants also emphasized the need for staff providing care to people with dementia to understand the potential impacts of different sub-types of dementia to enable post-diagnostic care to address individual needs (P8, P7). All of this expertise is crucial to ensure that older people with dementia, and particularly those with severe changed behaviors, receive skilled and responsive care, whichever setting they are in:

*What do we do for those people, those patients and where do they go, and who looks after them, and how do we look after those that look after them? (P8)*

### 3.2 Creating enabling and dementia-friendly environments

There were consistent references to the **challenges of hospital and nursing home care environments** for many people with dementia. Nursing participants described how loud, bright, crowded and busy care environments may trigger changed behaviors, and that standard care environments are characterized by comprising cramped spaces, many shared rooms, and lots of stimulation:

*[These environments] do not meet dementia-safe guidelines and do not support providing evidence-based care to people with dementia or cognitive impairment [or] changed behaviors at all. They don't support staff to do it safely. They don't support patients to have a great experience. (P7)*

Participants described how **enabling environments were key determinants of successful implementation of non-pharmacological interventions**. Participants across settings identified the importance of access to quiet, calm, low-stimulus spaces to avoid environmental triggers of changed behaviors. Suggested environmental assets included natural light, windows with garden outlooks, features of interest (pictures, books, wall art) and easily accessible outdoor spaces:

*So there are like different nooks and corners, and the lounges around the area, and also it has got a beautiful access to the courtyard. (P18)*

An important environmental feature was options to relocate people from communal settings for de-escalation, particularly if changed behaviors were disturbing other patients/residents, by using single or adjacent rooms, or other outdoor or quieter spaces (P7, P9, P23, P24). Providing security without feeling ‘locked in' was key in psychological safety needed to provide care to people with complex behavioral needs (P7, P24). Providing more home-like care and accommodation environments (P11, P2) including ‘village' models (P7 see above) were also identified as potential opportunities to better anticipating and responding to the needs of this population cohort (P4).

For escalation of care needs, where people with dementia have complex medical conditions, and/or have a need to be separated or secure from other residents due to risk of harm, there were **limited options that were fraught with competing priorities**. Existing dedicated space within the hospital environment to enable close observation and management of patients with complex needs (e.g., 4-bed areas with increased staffing and surveillance) was considered problematic if it limited the ability to separate patients who may trigger each other within the care environment and restricted access to outdoor spaces (P7, P5, P13). Infrastructure changes to better support effective “cohorting” or environmental grouping of people with dementia or other complex needs, in both hospitals and residential care, were suggested. This was argued to provide nurses with more options for early environmental and non-pharmacological interventions, and support better distribution of resources, including staffing, according to individual need. Emergency departments provide another instance where participants identified a need for a more dementia-friendly and secure space in this environment that allows for unobtrusive close observation of patients (P25, P26, P2, P17). In addition, accommodation options for people who may have higher care needs (extreme changed behaviors) were identified as a gap (2), where the only way to get intensive staffing ratios was via acute care, which was not an appropriate long-term care setting:

*We desperately need something that's in between a home environment and specialist unit (P2)*.

Participants highlighted that some nursing homes were choosing to reduce the size of their memory support units, effectively shrinking the availability of residential beds for people with complex dementia needs (P12, P12).

### 3.3 Appropriate prescribing in the context of physical and mental comorbidities

While all participants agreed that non-pharmacological interventions were important for managing changed behaviors, it was also widely acknowledged that pharmacological interventions are often required to address individuals' complex medical and mental health needs (P2).

A key example of complex medical needs was unmanaged pain as a significant and common trigger for changed behavior, with implications for access to effective assessment, pain relief and sustainable pain management plans (P2, P7, P15, P18):

*She is on her feet and screaming most of the time […] they [the community care home] were trying to get a pain patch from GP, [but] GP thinks there is no pain. [So] we commenced pain assessment …and medical team commenced on Norspan [pain relief] patch, and breakthrough, and she's more settled […] It's like a trial-and-error thing (P1)*

Appropriate medical prescription was seen as a critical part of the ‘toolkit' for providing care for people with complex presentations, including psychotic symptoms (P2, P9). Several participants suggested streamlining prescription guidelines for people with dementia to ensure consistency within and across settings while still allowing individual needs to be met (P2, P5, P7). The implementation of a more standardized approach would help increase consistency of care within inpatient settings and reduce the risk of inappropriate prescribing.

The lack of acknowledgment and appropriate **intersection between mental health and dementia services** was a concern for participants, where comorbidities and lack of clear documentation of diagnoses compounded the implications for medication prescription. Several participants indicated that there was often confusion about the most appropriate assessment, diagnosis and referral pathways: “*Look, I honestly think that people with dementia and psychotic symptoms just get shoved between “it's dementia, it's mental health”' (P2)*. There were also challenges in distinguishing between and responding to multiple comorbidities. For example, the appropriate use of antipsychotics in someone with dementia superimposed on a lifetime mental health diagnosis:

*All of those people saying ‘dementia doesn't need drugs'. No, dementia doesn't need antipsychotics, but mental health needs antipsychotics. So I feel like there's a really big delineation between the two that's not identified, not discussed enough. (P2)*

One participant also described that this confusion created a lack of understanding among some nursing homes about reporting requirements for antipsychotic use, resulting in people being denied or experiencing delays in receiving necessary medication:

*People just get so frightened that they're going to do the wrong thing or not report the right thing […] and actually that's resulting in people's human rights being violated almost because, you know, like some people won't be prescribed medications because it's like, oh, well, that's a restrictive practice or that's a chemical restraint. […] there's just a real misperception of what's actually being asked of people and it's really affecting the care that people are getting (P5)*.

To better support the intersection of multiple comorbidities that include mental health and dementia, staff recommended better **collaboration and shared care across geriatric and mental health** care settings. Integration of these services in acute settings was challenging where the availability of inpatient psychiatric beds for older people was limited and the available expertise in old age psychiatry and neuropsychology was already stretched (P22, P24, P12).

### 3.4 Safety and occupational violence as indicators for risk management

Episodes of severe to extreme changed behaviors involving acts of verbal and physical aggression or violence can result in mental and physical injuries to staff.

*As part of their worsening dementia […] they may be aphasic […] so it's really hard to communicate and they can't communicate to us what they really need […] they are communicating through physical aggression, unfortunately. (P1)*.

Participants provided several examples of physical and verbal aggression that they (or colleagues) have experienced while providing care to people with dementia experiencing severe changed behaviors (P25, P3, P26, P23, P5, P13). While staff may be aware and understanding of the impact of dementia on a person's ability to communicate and the underlying triggers for aggressive behaviors (P2, P1, P23), regular exposure to these situations has an impact on staff leave, burnout and turnover.

*I just get how you end up with that compassion fatigue when somebody's continuously trying to bite you, or hit you, or calling you every name under the sun. (P7)*

Within residential care settings, participants described how staff may be afraid of providing care to a person experiencing changed behaviors if they have past experience of being harmed (P18, P6) and that accessing additional timely external support is often challenging, triggering an ambulance transfer and hospital admission (P6). Some participants also expressed concern that nursing home staff may not have the same level of access to occupational violence training as staff employed in acute settings (P24), nor adequate access to diversional therapy services (P4)

*Occupational violence training, I don't think [nursing home staff] get any … so quite often they may respond to a situation and make it worse because they didn't know how to de-escalate. (P24)*

Participant references to occupational violence in their work were common, and associated with the complex nature of dementia symptomology and unmet needs (P25). Staff described avoidance of further escalation, and limited options for escalation (P1, P18), as well as cultural and racial abuse in relation to safety for staff (P5).

As mentioned previously, participants **recommended** a more proportionate “code gray” response to escalating, high-risk situations, rather than a “code black” response, providing an efficient, effective and less stressful experience for the patient and staff.

*We have instances where we wanted two wardsmen urgently to ensure staff and patient safety, due to responsive behaviors, but a Code Black brings seven to ten wards people, plus security staff, ten people coming into the room. That can be a traumatic experience for the patient (P1)*.

Participants described how failures to provide adequate staffing support pose safety risks for both patients and staff (See quotes in Theme 1), as well as the need for continuity of care where the staff'know' the person and how to best implement personalized de-escalation strategies based on personality and preferences (see quotes in Theme 5). In acute settings, nurses described the importance of having access to support from assistants in nursing (also often referred to as “specials”) and/or wards persons, particularly when occasions of personal care may be a trigger for changed behaviors and to support the implementation of behavior support plans. However, several nurses described that it was often difficult for them to access timely and appropriate additional support due to resource constraints and delays in actioning urgent support requests.

“*I think we often just say, well you know, we can just provide an assistant in nursing to help these people. But that's really not the case, and so it does become really challenging to try and advocate for the proper resources to be able to provide care to those people.” (P7)*

Reducing incidents of occupational violence for staff, particularly in service areas identified as having high levels of occupational violence, is an ongoing challenge that warrants consideration of recommendations highlighted by the expert staff experiencing the violence.

### 3.5 Early intervention and de-escalation, maximizing non-pharmacological interventions

Participants identified early intervention as one of the most effective ways to improve health services for people with dementia and to reduce the incidence and impact of severe changed behaviors (P14, P16, P25). Opportunities for early intervention were identified at different stages of an individual's care pathway and participants highlighted how seeking to intervene at each of the touchpoints in the health system could have a positive impact on an individual's health and care trajectories.

Early intervention begins with public health promotion to **destigmatise dementia** within the community and encourage people who have concerns about their cognition to seek a diagnosis.

*We're in the process of trying to normalize mental health. We should be trying to normalize dementia as well, and then perhaps that would actually allow people to not feel shame and be more proactive. (P21)*

Ensuring that people have timely access to diagnosis and related post-diagnostic information and multidisciplinary support is essential, so that individuals and families can better prepare for how a particular type of dementia may progress, and what may be the modifiable aspects of the disease progression (P8, P13).

*If you could have that MDT [multidisciplinary team] approach right when people are getting those diagnoses, there's so much potential for being able to put in that preventative support at that point in time. (P16)*

Participants also recommended **post-diagnostic rehabilitation programs** as an example of an early intervention to address the gap in post-diagnostic therapeutic support for people with mild-moderate dementia and their care partners (P16), as well as ensuring that people can access relevant support and services within the community to maximize wellbeing and avoid preventable hospital admissions (P14, P21). The role of discharging nurses or transitional services in identifying patients and care partners who may need additional support on discharge, referral to transitional outreach teams and the potential for follow up home visits were identified as other early intervention opportunities to reduce the risk of hospital readmission (P25, P17, P19). Additionally, encouragement to participate in advanced care planning and appointing an Enduring Power of Attorney (EPOA) in advance of a crisis situation were identified by participants as having multiple flow-on benefits for individuals, families and the health system more broadly (P8). Delays in hospital discharge are often related to the need to appoint an EPOA or guardian. These delays extended hospital stays, increasing risk of the patient acquiring hospital-related health complications and “*it just ends up being a bit of a treadmill of disasters*” (P7) that could be prevented with early intervention in the diagnosis, post-diagnosis and pre-crisis admission phases (P5).

**Person-centered care to prevent escalation in changed behaviors** was recommended by participants, but this relied on sufficient continuity of care staff to know the person with dementia and have the skills and knowledge to intervene:

“*Our staff here see things that happen, before it happens. Redirecting [people] and taking them outside […] if somebody gets cranky, if somebody gets upset […] you can see in their face, their expression, because you know them already you know when they're starting […] so you intervene, we have to be proactive.” (P9)*

Participants reported how high reliance on agency nursing staff in many residential settings, means that members of the care team often do not know the resident very well and do not understand or might not have the necessary experience to implement non-pharmacological approaches that might help diffuse difficult situations (P22, P24, P5).

**Occupational deprivation (i.e. the lack of meaningful activity)** was identified as a common trigger for escalation of changed behaviors and clinicians (across settings) identified the need to provide opportunities, and staffing, for people to participate in meaningful activities and increased social interactions to relieve boredom, frustration and loneliness (P16, P23).

*Residents are really bored. They've got nothing to do and they're just annoying each other, and it seems every single day it's escalating behavior and we've had a lot of people tell us, ‘you know, they just sit me in an exercise group and throw a balloon at me' and it's not stimulating. (P24)*

Recommendations for meaningful engagement ranged from improved training of care assistants and volunteers to activate engaging conversation and reminiscence therapy with people, to more complex arrangements involving access to outdoors, craft, music or physical activities relevant to the person's interest (P16, P1, P23, P16). Other recommendations included de-institutionalization of the care experience and involving people with dementia in meaningful daily activities such as meal preparation, reminiscence, and exercise activities appropriate to any given setting.

Participants highlighted recommendations to transform teams and work environments to nudge them toward better dementia practice, using evidence-based strategies for implementation such as: introducing a sunflower wall chart to encourage family members to share important information [State of NSW (Agency for Clinical Innovation), 2022]; comprehensive assessment; implementation of behavior support plans and related monitoring and documentation through the digital health record (P1).

### 3.6 Leadership of innovative and communicative transitional care services

Participants recommended that **cross-sectional leadership to enable early intervention at every potential stage** of health changes for people with dementia was crucial to addressing broader issues of dementia care, and particularly severe changed behaviors. Participants described the need to provide more community-based and in-home support services, given that most people with dementia are living in the community (P14, P23, P13).

Increasing awareness of and access to care pathways to support carers was seen as particularly important, with participants highlighting that navigating aged care, disability, health and community services can be incredibly challenging for carers:

“*She doesn't have the ability to, you know, go through the juggernaut of […] the zig zag of the services.” (P25)*.

Recommendations regarded integrated dementia care pathways, supporting carers to enable people with dementia to remain living at home (P23, P2, P17, P19):

“*[…] there's not the therapeutic supports, to support the capacity of the carer to support the capacity of the person with dementia […]. There's that sort of gap.” (P14)*

Participants reported the problems confronting carers and families included long wait times for Aged Care assessment, delays and difficulty in accessing home care support and other aged care, community and disability services, a lack of comprehensive early intervention supports and difficulties in accessing respite services, and this was worse for people experiencing moderate to severe changed behaviors (P19). Participants also described the need for **dementia and delirium nurse practitioners or clinical nurse consultants and case managers in the community** to provide timely and comprehensive assessment, and to help carers navigate pathways (P17, P13).

*[We need] nurse practitioners or clinical nurse consultants … in the community People [with dementia] end up in hospital all the time because if they've got delirium… and families don't know how to manage it and they just bounce in and out of hospital and there's just no need, it's distressing for them, a waste of resources Hospitals […are] awful for people with dementia (P10)*.

Clinical leadership, innovation and funding were recommended to overcome traditional silos and entrenched practices, to modify existing services to become more dementia inclusive, and create new services that are dementia-specific (P12, P14, P17, P12).

*I really think it's time to be creative with our ecosystems and think outside the box (P2)*.

Leadership decisions were highlighted regarding advocacy for allocating resources for workplace education and training, for staffing levels and skill mix, for making environmental changes, or for influencing discharge decisions. Hospital-based and transitional participants also discussed needing to address bias in the residential care sector, where patients wouldn't be considered for transition if a dementia service was involved, because that meant that changed behaviors may be part of the complex needs (P1, P5, P15, P12).

*I referred a patient to DSA [Dementia Services Australia] for transitional support and when they contacted the nursing home, the nursing home was like, ‘Oh, [that] means the patient is having behaviors. Then probably we won't be able to take the patient'. […] it's like, you know, like a stigma […]. I don't want [people] getting discriminated [against] because DSA are involved (P1)*

Participants considered the establishment of two specialist dementia units in the community had helped reduce the number of people with severe changed behaviors being “stuck” in hospital for very extended periods of time. However, it had not solved the issue with some patients being returned to acute care for other issues, and it not providing long term care solutions (P5, P7, P5).

Staff were very positive about the valuable role of **geriatric in-reach services (to community and residential care)** (P13, P15), in reducing ambulance and emergency department usage. Without these services, there were minimal avenues to escalate care (P24). While these hospital-based in-reach services are not dementia-specific, a significant proportion of their clients are living with cognitive impairment or a diagnosis of dementia, and additional investment to maximize the impact of these specialist staff would help address the care needs of people living in the community and nursing home care who are experiencing changed behaviors (P14, P20 & P24).

Communication and collaboration across sectors to reduce service silos were repeatedly highlighted as recommendations. Participants identified the need for continuity of care for people with dementia within the health, aged and disability systems, but indicated that this was often difficult to achieve due to the siloed nature of many services and funding models (P13, P3). Participants from both nursing home and acute settings highlighted the need for increased trust and transparency to facilitate discharge of patients from hospital into suitable residential accommodation (P1).

“*I think building that trust in relationship between the acute sector and the homes is really important and having that two-way communication.” (P7)*

Residential care participants expressed concerns that they are not always given accurate information about a person's care needs; they feel they lack the internal capacity to meet a person's care needs; and they do not feel confident that they have timely access to necessary additional (in-reach) support to meet a person's care needs (P2, P6, P4, P6). From the other perspective, ambulance and emergency staff struggle to obtain sufficient information or complete assessments to determine appropriate admissions (P15). Increasing levels of transitional support and adopting a person-centered partnership approach to enable this cohort of patients to successfully transition into residential care were identified as urgent priorities (P3, P5, P7). The establishment of a tri-service crisis emergency out-reach response involving ambulance, police and gerontological/mental health nursing expertise was recommended to support escalation of care in changed behaviors (P24).

The movement of staff between different teams within an organization was identified as a catalyst for the “new host team” to gain an understanding of “*how it works over there*” (P8). Staff rotations or secondments to different teams were recommended to increase knowledge sharing within organizations, understanding service roles and referral pathways, providing professional development opportunities, and helping to address staff burnout (P12). Another opportunity for greater collaboration identified by participants was enabling professional supervision and mentorship that are not based on disciplinary models to expand professional networks and provide “*a space where you could talk about how to manage these behaviors”* (P8) and connect with colleagues who have expertise and skills that would be helpful.

## 4 Discussion

This research provides a constructive lens on dementia services across settings, which used the experiences and recommendations of expert multidisciplinary clinicians from multiple care types providing dementia care across a single jurisdiction. Each of the participants' perspectives was heavily affected by their position within the system, and the lens through which they perceived the rest of the system, and where gaps and opportunities existed for improved pathways of care, workforce development and health service responsiveness. These findings provide a unique, multidisciplinary and multi-service settings perspective compared to previous research on staff experiences of dementia services (e.g., Borbasi et al., 2006; Tropea et al., 2017), in that they are cross-sectional and emphasize potential solutions within and across services. The findings highlight that, at worst, people with dementia are presenting in crisis states where all social capital has been exhausted for simple needs such as pain that have escalated without effective treatment, and services have difficulty in assessing, treating and referring onwards. In the best cases, people with dementia and their carers are supported early with post-diagnostic support, maximized in cognitive, physical and social strengths, and are better able to interact with the health services available to them.

A key finding was the need to **bolster community capability** across services, settings and workforce for timely and comprehensive responses to health needs of people with dementia and their carers at any stage of diagnosis and care. Participants emphasized early investment in whole of community education and access points, with a ‘no wrong doorway' approach to improve entry to diagnosis, post-diagnostic support, crisis intervention, care transitions, and escalation of clinical and/or behavioral care.

Effective post-diagnosis rehabilitation and reablement programs were promoted by participants for dementia-friendly community intervention. These have been demonstrated to have high satisfaction and adherence, with positive functional and cognitive outcomes for people with dementia and care partners (D'Cunha et al., 2023, 2024, 2025a; Chelberg et al., 2025) and align with **dementia-friendly services** recommendations (Low et al., 2021). While there is recent literature on post-diagnosis rehabilitation and reablement programs in Australia (D'Cunha et al., 2025a; Chelberg et al., 2025; Jeon et al., 2025; Clemson et al., 2021), there is a gap between recommendations and current practices in Australia (Mulhall et al., 2026). For example, Cognitive Stimulation Therapy is recommended in several clinical guidelines for mild-to-moderate dementia, including the National Institute for Health and Care Excellence (NICE) guidelines in the United Kingdom (National Institute for Health and Care Excellence, 2018). The Australian Dementia Clinical Practice Guidelines and Principles of Care are currently being updated, with the draft recommending ‘to consider offering structured and targeted interventions with a focus on activity, participation, and interpersonal relationship-building' to address changed behavior (Monash University, 2026).

As part of an inclusive access approach, streamlined emergency assessment and treatment were repeatedly argued by participants as key to effective assessment, hospital avoidance, and high-quality care throughout different stages of dementia. These included a range of transitional (between services) models, also variously referred to as out-reach or in-reach in the literature (where health staff, most often nurses, ‘reach out' from hospitals into nursing homes or the community). A recent scoping review found transitional models of care were associated with improved health outcomes for people with dementia, including fewer emergency presentations, shorter length of stay and lower costs, reduced hospital admissions and medication use, greater use of non-pharmacological interventions, reduced carer burden, and increased documentation of dementia diagnosis (Mete et al., 2025). Emergency department streamlining, sometimes with a phone liaison with nursing homes, and intensive behavior support units were associated with positive health and service outcomes for dementia care (Mete et al., 2025). Previous research has found that patient outcomes improved with skilled multidisciplinary gerontology emergency department teams, such as the Geriatric Emergency Department Intervention (GEDI) (Marsden et al., 2018, 2020; Wallis et al., 2018). Nurse practitioners in outreach roles have been found effective in diagnosing and documenting a dementia diagnosis, including during their training or transition to fully qualified nurse practitioner (Pond et al., 2021). In other research, transitional nurse practitioner or advance practice nurse-led models of care were noted to contribute to decreased hospital admissions and/or length of stay (Borbasi et al., 2010; Hullick et al., 2016; Kwa et al., 2021; Dwyer et al., 2017; Craswell et al., 2020) and decreased length of emergency admission (Hullick et al., 2016). Funded transitional positions to support advanced practice nurses to work while they advance their study may be important in areas that have workforce shortages.

The second key finding of this research was the emphasis on **knowledgeable care**, with dementia-friendly and dementia-inclusive services. Services need to be staffed by clinicians with generalist gerontological awareness, skills and knowledge including delirium, pain assessment in cognitive impairment, de-escalation and mental health skills, application of reablement approaches for people with dementia and trauma-informed approaches. Getting increased gerontological expertise to the moments of assessment and intervention is key, and in particular non-pharmacological evidence-based approaches (See Supplementary 1, created for the co-funder). Current research indicates the health workforce is not well prepared for this skill set, despite the growing population of older people (Annear, 2020; Atee et al., 2021a; Bail and Grealish, 2016; Bianchi et al., 2024; Cations et al., 2020; Fisher et al., 2025; O'Connor et al., 2020). Professional pathways of education and mentoring, to support clinicians to grow from generalist gerontological knowledge to specialist knowledge was highlighted for all disciplines from GPs, geriatricians, nurses, allied health and other health workers. This ‘gerontologising of the workforce' has been mentioned in relation to other specialties (Luke and Beck, 1999), but not the health workforce as a whole, and is highlighted by these participants as crucial because people with dementia will be cared for in all sections of the health service, not just specialist ones. The lack of dementia expertise in residential settings and acute non-geriatric settings is concerning but may be addressed through workforce education initiatives, which may also relate with staff turnover (Traynor et al., 2025). Importantly, these participants emphasized the inequality in **workloads and staffing numbers for people with dementia** in hospitals and aged care settings compared to other sub-specialties. This highlights that it is not the workforce shortages that are so frequently blamed in aged care issues-rather, the complexity of the work is not recognized, recompensed nor rostered for (Thwaites et al., 2023; Morioka et al., 2025; Simmons et al., 2025). For example, participants described episodes of occupational violence as a normal and barely remarkable incidence; seeking to normalize and humanize the response for the person with dementia, they minimized the impact of these situations to themselves while emphasizing the need to keep patients/residents safe. They also reported needing to continually challenge assumptions regarding the perceived “basic care” of older person nursing, highlighting that providing simple interventions for complex people in complex settings needs advocacy (Bail and Grealish, 2016). Research in “acute care of the elderly” finds increased registered nurse staffing is associated with decreased complications (Morioka et al., 2025; Simmons et al., 2025).

**The third key finding is in relation to the** high-level specialist services proposed by the interviewees included tri-response mental health crisis models, which have been shown to be effective in responding to crisis (Evangelista et al., 2016), and are a key area for further development in responding to population changes and the increasing number of people with dementia being attended by police (Ries et al., 2023). Carer distress and respite needs are often a key area of need in relation to these crisis situations and so respite accommodation and care is a necessary component (Herron and Runacres, 2023; Oliveira et al., 2019). The participants in this study add that suitable “cohorting” options (environmental grouping of patients to maximize staffing effectiveness) in all settings are necessary to enable effective matching of personalities and behavioral expressions within close-quarters living to reduce triggers for behavior escalation. Future research and building design need to consider spaces and options for de-escalation, living spaces that can separate people when other people are the behavioral triggers, and relevant to all settings and kinds of care including acute for clinical care, and residential for respite or permanent care. This finding has been illuminated elsewhere but with a focus on “cohorting” in order to simplify staffing to manage increased observation needs (Bail et al., 2023; Handley et al., 2023). In particular this is important in relation to how p**erceptions of severity of changed behaviors** can be **context-specific**. For example, “wandering” may be considered problematic and a “behavior” if the person is in a place where there is no safe space to walk. The elements interpreting “dignity of risk” in all health settings in relation to environmental resources, and the supportive workforce skills in supported decision making and behavior support plans, will be important for these services (Foundas, 2025). More research on cohorting approaches to better manage de-escalation is needed, with a focus on impacts on resident and staff outcomes.

To better **visualize the findings and recommendations**, we refer to Brodaty et al. (2003) seven-tiered triangular model to assist in dementia care service delivery planning, specifically for the care needs of people with dementia experiencing changed behaviors. A key element of this model was the emphasis on a system-wide preventative approach with investment in early intervention as a component of integrated care pathways for people with dementia to minimize demand and need for higher levels of service (Brodaty et al., 2003). Based on the participant recommendations, we propose a revised model of the triangle to better visually represent the accumulation of interventions, services and workforce to better respond to all stages of dementia in the community, residential and acute care that has a flow-on effect upon dementia progression, as well as workforce development to create a responsive health system for people with dementia.

**Figure 1:**
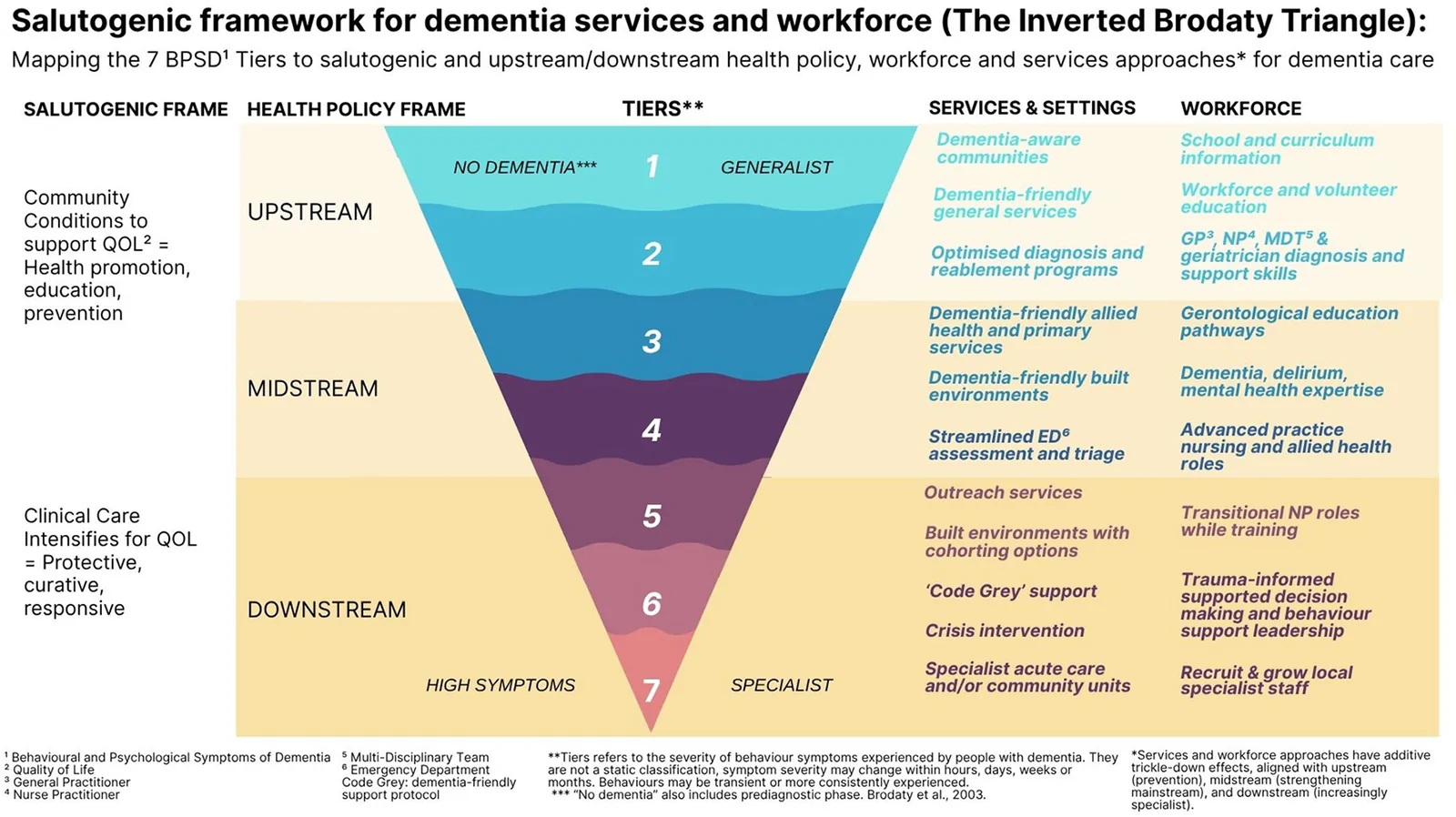
Salutogenic framework for dementia services and workforce.

The broad-based dementia-friendly services can be seen to match Tiers 1 and 2, with mid-level knowledgeable care (e.g., Tiers 3, 4, and 5) and high-level specialist services (e.g., Tiers 6 and 7). The “inverted Brodaty triangle” (hereafter referred to as the Salutogenic Triangle), presented in Figure 1, is dually informed by the theories of “upstream/downstream” health policy services approach (Perry et al., 2022), and the salutogenic approach (Ehk et al., 2024; Golembiewski and Zeisel, 2022). The health policy approach emphasizes interventions for community and societal needs to have broad community impact. These interventions are preventative and prophylactic and impact “downstream” to improve health outcomes for individuals within that population. These approaches have traditionally been used for disease prevention rather than symptom amelioration (Perry et al., 2022). A complementary but philosophically unique theory is the salutogenic approach which focuses on “wellbeing” as a strengths-based approach, rather than pathogenic approaches that focus on “disease” and a deficit-approach, and uses a figure of “health in the river of life” to communicate (Lindstrom and Eriksson, 2010). The “health in the river of life” emphasizes health promotion and health education, preventative and protective interventions to promote quality of life, with inclusion of curative approaches when applicable (Lindstrom and Eriksson, 2010). This emphasis de-medicalises health conditions and empowers living well, acknowledging that illness, disease and risks are part of living, but that life is the primary force to be responded to. This similarly emphasizes “gerontology” (a multidisciplinary approach to aging) rather than solely “geriatric” (medical treatment of diseases).

The “Salutogenic approaches to dementia services and workforce” Triangle figure emphasizes the clinician recommendations for investing in UPSTREAM **broad-based dementia-friendly community services** (e.g., generalized dementia knowledge in all clinicians, post-diagnostic multidisciplinary care teams focussed on enablement), MIDSTREAM **enabling mid-level knowledgeable care** (e.g., hospital avoidance and residential in-reach services and gerontological nurse leadership in emergency departments to streamline assessments), and DOWNSTREAM **high-level specialist services** for intensive support for those who need it when they need it (crisis intervention, purpose-built units in hospitals and residentially).

Using the salutogenic frame, the emphasis of the figure is on more services for more people early in the diagnosis pathway to reduce acceleration and proliferation of cases to high-consequence behavior changes, and a trickle-down effect for gerontological awareness and expertise for the health workforce. The figure promotes early escalation of response within mainstream services, which may prevent deterioration to severe symptoms. Downstream services of increasingly specialist services are shown as essential, in that no matter how much education or dementia friendly design is provided, no mainstream worker or service are adequately equipped to support the severe tiers of behavioral presentation.

This approach is consistent with literature that highlights that supporting care partners and maintaining the mobility of the person with dementia are associated with decreased risk of nursing home admission (Toot et al., 2017), sustained maintenance of functional and cognitive status (Draper et al., 2018) (i.e., reduce risk of decline), and increased carer wellbeing and carer health outcomes (D'Cunha et al., 2023, 2024). Using the salutogenic frame, the figure demonstrates how strengthening and investing in prevention and mainstream supports can provide cumulative benefits across the lifespan of individuals with dementia as well as the communities they live in.

## 5 Limitations and future directions

This research has highlighted potentially modifiable components of care that can be changed to improve the care of people with dementia. This study is limited in that it does not include people with dementia, nor distinguish between different types of dementia or unique and diverse needs of population groups. It is also limited by a larger sample of nurses compared to medical and allied health perspectives, though not in dissimilar proportions to the population, but missing general practitioner perspectives. The study was conducted in a single urban center and therefore does not include the perspectives of clinicians and service providers working in rural and regional areas of Australia. Similar research in other major urban settings as well as work incorporating regional and remote service providers would be a valuable area for future research (Gulline et al., 2025). The strength of this paper is the unique multi-sector perspective responding to the important challenge of workforce, providing a visualization of expert recommendations for sustainable salutogenic approaches that may improve staff retention and satisfaction with quality of care, with the enablement of ecosystems across interfaces of care of community, acute and residential settings.

## 6 Conclusion

Our communities are aging, and dementia experience will increase for individuals, and we cannot keep relying on the sheer good will and dedication of staff. In coming years, demand for dementia care services will increase and there is growing recognition that community, health, disability, rehabilitation, aged care and palliative services will need to adopt new approaches to better address the diverse and often-complex care needs of people with dementia experiencing changed behaviors; this new inverted model builds on theory, practice and research to promote sustainable salutogenic approaches to dementia services.

All too often in health service responses to dementia, the focus is on the long lengths of stay and the behavioral and psychological symptoms of dementia, and particularly the high end of changed behaviors (e.g., Möllers et al., 2019). The Salutogenic Triangle attempts to improve the visual representation of the intended person-centered emphasis of the original model regarding services tiers to respond to dementia. In this way, policy makers, service providers and clinical leaders can focus on and invest in preventative and supportive interventions that can help decrease the incidence and severity of changed behaviors through broad-based community awareness and dementia-friendly health environments, midstream targeted early intervention strategies, and downstream high-level intensive services for specific dementia care needs.

## Data Availability

The raw data supporting the conclusions of this article will be made available by the authors, without undue reservation.
