# Supplementary material for: Inverting the ‘dementia triangle' as a salutogenic approach to avoiding the “treadmill of disasters” in changed behaviors: a qualitative study
Source: Front Dement. 2026 Sep 8;5:1891190. doi: 10.3389/frdem.2026.1891190 (PMC13593413; doi:10.3389/frdem.2026.1891190)
Supplement: Supplementary file 1 [file Data_Sheet_1.pdf]

UNIVERSITY OF CANBERRA | CENTRE FOR AGEING RESEARCH AND TRANSLATION

EVIDENCE-BASED INTERVENTIONS FOR PEOPLE LIVING WITH DEMENTIA AND THEIR CARERS

Cognitive Stimulation Therapy

The intervention enhances cognitive function, language, quality of life, communication, mood, and daily functioning in individuals with dementia while also reducing depression and behavioural changes[1,2,3,4,5,6] . For carers, CST is associated with lower stress and anxiety levels and better quality of life.

Care partner education

Tailored interventions that enhance skills and knowledge of care partners in managing dementia-related challenges reduce behavioural changes, depression, anxiety, and caregiver burden while improving quality of life and wellbeing[7,8,9,10,11].

COPE Program

Care of the Older Person with dementia in their Environment program has been shown to reduce functional dependence and enhance activity engagement in individuals with dementia and improve overall well-being and coping in carers[12,13,14].

Physical activity

Targeted physical activity is the optimal treatment for dementia as it reduces neuropsychiatric symptoms, lowers caregiver burden, and enhances the quality of life for both individuals with dementia and their carers[15].

Non-pharmacological interventions

Non-pharmacological interventions reduce changed behaviours and are effective for overall symptoms, depression, and agitation and improve carer reactions to changed behaviours[16,17]

Meaningful engagement

Engagement in meaningful activities (such as reminiscence, volunteering, chores) was significantly associated with decreased odds of having agitation in people with dementia[24]

Reablement interventions

Increased participation in activities of daily living, quality of life, physical function, reduction in use of home and health care services. Supports carers by reducing stress and enhancing communication enabling better support[22].

Tailored dietary advice

Dietary strategies improve cognitive function, physical health, and hydration by implementing regular monitoring, personalised plans, and educating carers about mealtime challenges and nutritional support[18,19].

Increase staffing

Low staff-to-patient ratios are associated with increased changed behaviours [20].

Prevent delirium

Prevention of delirium in people with dementia reduces complications and further cognitive decline [15].

Correct sensory loss

Effective management of sensory loss using hearing aids, glasses, assistive listening devices, and speech therapy can improve symptoms of dementia[21].

Minimise antipsychotic medication

Antipsychotic medication should never be used as a first response, and increases risks of hip fracture, stroke and death. Overuse of antipsychotics indicates a lack of staff with specialised training and implementation of person-centred care plans[20,23].

References: 1. Xiang C, Zhang Y. Comparison of cognitive intervention strategies for individuals with Alzheimer's disease: a systematic review and network meta-analysis. Neuropsychology Review. 2024;34(2):402-16.; 2. Woods B, Rai HK, Elliott E, Aguirre E, Orrell M, Spector A. Cognitive stimulation to improve cognitive functioning in people with dementia. Cochrane Database Syst Rev. 2023;1(1):CD005562; 3. Paggetti A, Druda Y, Sciancalepore F, Della Gatta F, Ancidoni A, Locuratolo N, et al. The efficacy of cognitive stimulation, cognitive training, and cognitive rehabilitation for people living with dementia: a systematic review and meta-analysis. Geroscience. 2024; 4. Saragih ID, Tonapa SI, Saragih IS, Lee B-O. Effects of cognitive stimulation therapy for people with dementia: A systematic review and meta-analysis of randomized controlled studies. International Journal of Nursing Studies. 2022;128:104181; 5. Wang YY, Yang L, Zhang J, Zeng XT, Wang Y, Jin YH. The Effect of Cognitive Intervention on Cognitive Function in Older Adults With Alzheimer's Disease: A Systematic Review and Meta-Analysis. Neuropsychol Rev. 2022;32(2):247-73; 6. Watt JA, Goodarzi Z, Veroniki AA, Nincic V, Khan PA, Ghassemi M, et al. Comparative efficacy of interventions for reducing symptoms of depression in people with dementia: systematic review and network meta-analysis. BMJ. 2021;372:n532; 7. Meng X, Su J, Li H, Ma D, Zhao Y, Li Y, et al. Effectiveness of caregiver non-pharmacological interventions for behavioural and psychological symptoms of dementia: An updated meta-analysis. Ageing Research Reviews. 2021;71:101448; 8. Sun Y, Ji M, Leng M, Li X, Zhang X, Wang Z. Comparative efficacy of 11 non-pharmacological interventions on depression, anxiety, quality of life, and caregiver burden for informal caregivers of people with dementia: A systematic review and network meta-analysis. International Journal of Nursing Studies. 2022;129:104204; 9. Wiegelmann H, Speller S, Verhaert L-M, Schirra-Weirich L, Wolf-Ostermann K. Psychosocial interventions to support the mental health of informal caregivers of persons living with dementia – a systematic literature review. BMC Geriatrics. 2021;21(1):94; 10. Bushell D, Jones C, Moro C. The effectiveness of educational interventions in the community that aim to improve informal carers knowledge of dementia anatomy, physiology, progression, and impact on behavior: a systematic review. Front Dement. 2023;2:1156863; 11. Birkenhäger-Gillesse EG, Achterberg WP, Janus SIM, Kollen BJ, Zuidema SU. Effects of caregiver dementia training in caregiver-patient dyads: A randomized controlled study. Int J Geriatr Psychiatry. 2020;35(11):1376-84; 12. Gitlin LN, Winter L, Dennis MP, Hodgson N, Hauck WW. A Biobehavioral Home-Based Intervention and the Well-being of Patients With Dementia and Their Caregivers: The COPE Randomized Trial. JAMA. 2010;304(9):983-91; 13. Clemson L, Laver K, Rahja M, Culph J, Scanlan JN, Day S, et al. Implementing a Reablement Intervention, "Care of People With Dementia in Their Environments (COPE)": A Hybrid Implementation-Effectiveness Study. The Gerontologist. 2021;61(6):965-76; 14. Fortinsky RH, Gitlin LN, Pizzi LT, Piersol CV, Grady J, Robison JT, et al. Effectiveness of the Care of Persons With Dementia in Their Environments Intervention When Embedded in a Publicly Funded Home- and Community-Based Service Program. Innovation in Aging. 2020;4(6):igaa053; 15. Livingston, G., Huntley, J., Liu, K.Y., Costafreda, S.G., Selbæk, G., Alladi, S., Ames, D., Banerjee, S., Burns, A., Brayne, C. and Fox, N.C.(2024). Dementia prevention, intervention, and care: 2024 report of the Lancet standing Commission. The Lancet, 404(10452), 572-628; 16. Meng, X., Su, J., Li, H., Ma, D., Zhao, Y., Li, Y., & Sun, J. (2021). Effectiveness of caregiver non-pharmacological interventions for behavioural and psychological symptoms of dementia: An updated meta-analysis. Ageing Research Reviews, 71, 101448; 17. Yin, Z., Li, Y., Bao, Q., Zhang, X., Xia, M., Zhong, W. & Liang, F. (2024). Comparative efficacy of multiple non-pharmacological interventions for behavioural and psychological symptoms of dementia: a network meta-analysis of randomised controlled trials. International Journal of Mental Health Nursing, 33(3), 487-504; 18. Mole L, Kent B, Abbott R, Wood C, Hickson M. The nutritional care of people living with dementia at home: A scoping review. Health Soc Care Community. 2018;26(4):e485-e96; 19. Volkert D, Beck AM, Faxén-Irving G, Frühwald T, Hooper L, Keller H, et al. ESPEN guideline on nutrition and hydration in dementia - Update 2024. Clinical Nutrition. 2024;43(6):1599-626; 20. Sibley, E., & Mercer, C. (2023). Management of behavioural and psychological symptoms of dementia (BPSD) An integrative review. Kaitiaki Nursing Research, 14(1), 41-49; 21. Armitage, Christopher J., Leroi, Iracema and Millman, Rebecca E. (2022) Effectiveness of hearing rehabilitation for care home residents with dementia: a systematic review. Journal of the American Medical Directors Association, 23 (3). pp. 450-460; 22. Rahja, M., & Haddock, R. (2021). Reablement interventions for community dwelling people living with dementia; 23. Mok, P. L., Carr, M. J., Guthrie, B., Morales, D. R., Sheikh, A., Elliott, R. A., Camacho E.M., van Staa T., Avery A.J.(2024). Multiple adverse outcomes associated with antipsychotic use in people with dementia: population based matched cohort study. bmj, 385. 24. Klinedinst, N. J., Holmes, S., Resnick, B., & Galik, E. (2024). The relationship between perceived engagement in meaningful activity and behavioral and psychological symptoms of dementia among assisted living residents. Geriatric Nursing, 60, 528-532.
